# Actin remodeling by human RNA virus: mechanisms and targeted intervention

**DOI:** 10.3389/fcimb.2026.1975477

**Published:** 2026-10-22

**Authors:** Jie Gao, Wei Yu

**Affiliations:** 1 Institute of Biochemistry, College of Life Sciences and Medicine, Zhejiang Sci-Tech University, Hangzhou, China; 2 Zhejiang Provincial Key Laboratory of Silkworm Bioreactor and Biomedicine, Hangzhou, China

**Keywords:** actin recombination, actin regulators, human RNA viruses, intervention strategies, viral infection

## Abstract

The actin cytoskeleton is a central dynamic network in eukaryotic cells which preserves morphology, motility, and intracellular transport. Extensive research has confirmed the importance of actin in RNA viral infection. Actin remodeling, as a core host mechanism exploited by RNA viruses, strongly affects viral replication and transmission efficiency. This article reviews the key regulatory molecules involved in actin dynamics. Based on this foundation, we focus on recent advances in the molecular mechanisms by which six representative human RNA viruses(severe acute respiratory syndrome coronavirus-2, influenza A virus, respiratory syncytial virus, dengue virus, ebola virus and human immunodeficiency virus) hijack the host actin network, revealing the actin hijacking pathways that RNA viruses tend to select. Finally, we further summarize the current status of research on antiviral compounds that target the actin signaling pathway. Given that targeting host factors offers the unique advantage of broad-spectrum antiviral activity, this strategy offers promise for overcoming drug resistance caused by high viral mutation rates. However, its clinical application still faces difficulties in balancing cytotoxicity and efficacy. This article intends to provide a conceptual framework for novel antiviral intervention approaches targeting actin.

## 1 Introduction

The cytoskeleton is mainly composed of microtubules, actin filaments, and intermediate filaments. Actin is a widely distributed and conserved protein in eukaryotes, existing in cells in two forms: globular actin (G-actin) and filamentous actin (F-actin) (Pollard, 2016). F-actin is the primary form through which actin exerts its biological functions; it not only constitutes the cytoskeleton to support the cell’s basic morphology but also participates in various cellular activities such as cell division, endocytosis, exocytosis, and vesicular transport (Bibeau et al., 2020; Wu and Chan, 2022). Furthermore, extensive research has demonstrated that actin reorganization plays an irreplaceable part in viral infection (Taylor et al., 2011; Liao et al., 2018).

Actin polymerization, depolymerization, and spatial organization don’t just happen by chance—they’re tightly controlled by signaling modules that evolution has kept around for good reason. Actin-binding proteins (ABPs), stand out here. They serve as the main players when it comes to regulating actin dynamics. Actin assembly proceeds efficiently through the coordinated action of ABPs with diverse functions, such as the actin-related protein 2/3 (Arp2/3) complex and cofilin (Moon et al., 1993; Welch et al., 1997). The activation of ABPs cannot occur autonomously, requiring​ stepwise transduction of upstream signals through multiple levels. Among these, the three major subtypes of the Rho GTPase family—Ras-related C3 botulinum toxin substrate 1 (Rac1), cell division control protein 42 homolog (Cdc42), and Ras homologous A (RhoA)—serve as the central molecular switches linking upstream signals to cytoskeletal effector.

proteins (Ridley and Hall, 1992; Ridley et al., 1992; Nobes and Hall, 1995). These three classes of GTPases differentially regulate ABP activity through their respective downstream effector molecules, thereby mediating the assembly and remodeling of lamellar pseudopods, filopodia, and stress fibers, respectively (Nobes and Hall, 1995). ABPs and Rho GTPases act together as key regulators of actin kinetics and play a vital role in the actin regulatory network.

Numerous studies have shown that viruses can use actin to facilitate infection (Cudmore et al., 1995; Lehmann et al., 2005; Yoder et al., 2008). Unlike DNA viruses, RNA viruses are microorganisms that use RNA as their genetic material and are obligate intracellular parasites (Baltimore, 1971; Nie et al., 2023). With the frequent outbreaks and spread of various human pathogenic RNA viruses in recent years, including severe acute respiratory syndrome coronavirus-2 (SARS-CoV-2), influenza A virus (IAV), respiratory syncytial virus (RSV), dengue virus (DENV), ebola virus (EBOV) and human immunodeficiency virus (HIV), the formulation of targeted antiviral agents has become an urgent priority (Roychoudhury et al., 2020). Against this backdrop, the close interaction between viruses and the host actin cytoskeleton has appeared as a highly promising new therapeutic target. In recent years, the molecular mechanisms by which RNA viruses regulate the host actin cytoskeleton to facilitate processes such as invasion, replication, and release have gradually been elucidated, becoming one of the hot topics in virology research. The utilization of actin by RNA viruses such as SARS-CoV-2 occurs during multiple stages of cellular infection, making them more representative and cutting-edge in the field of virology. Compared to DNA viruses, RNA viruses often hijack the actin cytoskeleton in more unique and diverse ways. The cytoplasmic replication characteristic of RNA viruses makes their dependence on the actin cytoskeleton more direct and comprehensive. They achieve spatiotemporal remodeling of the cytoskeletal structure by finely regulating Rho GTPase molecular switches and specifically hijacking actin-binding proteins (ABPs) to meet the demands of different stages of infection. Furthermore, infection by some viruses simultaneously activates the host’s countermeasures. For example, IAV infection induces filopodia formation, but at the same time, the host suppresses filopodia to block viral entry (Hunziker et al., 2022).

In addition to promoting infection, dysregulation of the actin regulatory axis has also been found to contribute to pathological phenotypes associated with RNA viruses, such as co-aggregation and disruption of the immune barrier, among others (Duan et al., 2026; Dunn et al., 2026). However, compared to its role in the viral infection cycle, research concerning the mechanisms underlying actin dynamics in pathological phenotypes remains relatively limited; this review provides only a brief discussion of this topic in relevant viruses.

This article reviews recent advances in the molecular mechanisms by which various important human RNA viruses use actin to facilitate infection. In addition, this article outlines antiviral intervention approaches targeting distinct actin pathways, intending to give insights into developing antiviral approaches against RNA viruses.

## 2 Key regulatory molecules in actin kinetics

Actin assembly is a highly dynamic cycling process. First, actin monomers undergo nucleation to form a stable trimeric core (Pollard, 1986). Subsequently, ATP-G-actin preferentially binds to the fast-growing end of the core, known as the plus end. As the actin filament continues to extend, the ATP on G-actin is hydrolyzed to ADP, causing a decrease in the local stability of the filament (Carlier, 1990). Ultimately, the ADP-actin subunits detach from the slow-dissociation end, or the negative end. The detached monomers are subsequently recycled. The recycled monomers are reloaded with ATP and enter the next round of the polymerization cycle (Mockrin and Korn, 1980). This continuous process, in which positive-end polymerization is coupled with negative-end depolymerization, forms the basis of cytoskeletal plasticity. In addition, assembled actin filaments often need to be cross-linked to form robust, functional actin structures (Hartwig and Stossel, 1975; Bretscher, 1981). The efficient execution of these processes typically requires the participation of various ABPs with distinct functions, as well as their upstream regulatory switches, the Rho GTPases (see **Figure 1**).

**Figure 1:**
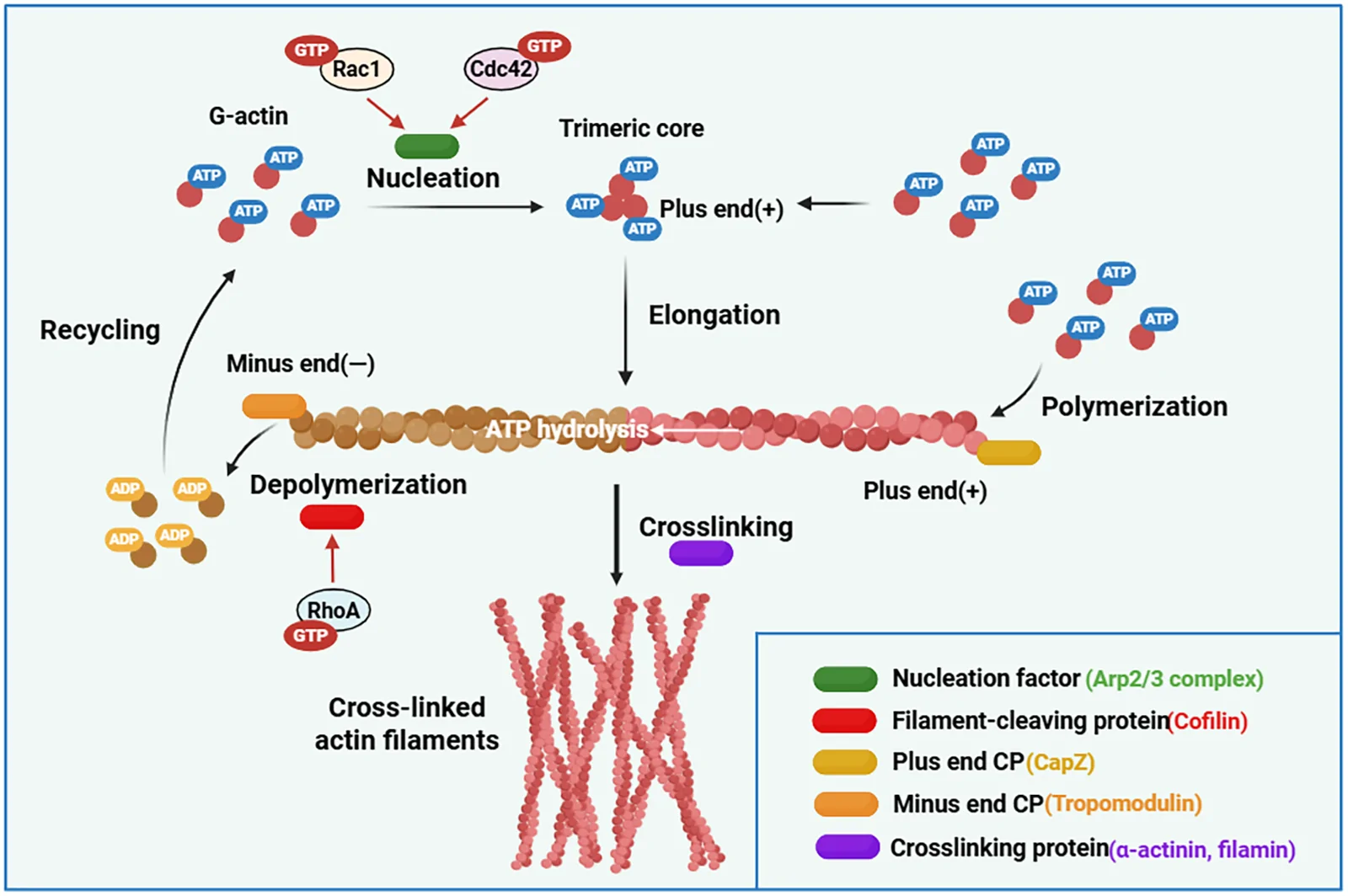
The basic process of actin assembly. The ATP-bound form of G-actin is catalyzed by nucleation factors, such as the Arp2/3 complex, to form a trimeric core, after which the extension of the actin filament at the plus end begins. At this point, ATP-bound G-actin is continuously added to the plus end, driving actin polymerization. Once the actin filament has grown to a certain length, the ATP bound to G-actin is hydrolyzed to form ADP, and the minus end of the actin filament undergoes depolymerization catalyzed by the filament-cutting protein cofilin. The ADP-bound G-actin is then released and will be converted back to ATP-bound G-actin to play its role when the next round of polymerization begins. To ensure proper assembly, the plus and minus ends of actin filaments bind to specific CPs. The plus end of an actin filament typically binds to CapZ, while the minus end binds to tropomodulin. Upon completion of assembly, actin filaments cross-link to form cross-linked actin filaments with the participation of cross-linking proteins such as α-actinin and filamin. Rac1 and Cdc42 primarily act on the nucleation factor Arp2/3 complex, while RhoA mainly regulates the filament-cutting protein cofilin. This image shows only representative regulatory factors of actin assembly; other regulatory factors are described in the main text. See the list of abbreviations at the end of the paper for the full forms of all abbreviations. Created in BioRender. Jie, G. (2026) https://BioRender.com/nup1kv6.

### 2.1 ABPs

ABPs are a class of molecules that act directly on actin. They are commonly categorized into functional groups such as nucleation, cleavage, capping, and cross-linking to regulate actin (Pollard, 2016). Nucleation factors such as the Arp2/3 complex primarily promote actin nucleation (Welch et al., 1997; Amann and Pollard, 2001). Cofilin is considered the primary filament-cleaving protein responsible for cleaving old filaments to facilitate microfilament disassembly (Maciver et al., 1991). Capping proteins(CPs) bind to and cap the plus or minus ends of actin filaments, terminating filament growth. Positive-polarity CPs, such as CapZ, can prevent excessive actin filament elongation by binding to the plus ends of actin filaments (Schafer et al., 1994). Tropomodulin is a classic negative-end capping protein that prevents excessive depolymerization of the negative ends of actin filaments (Weber et al., 1994). Proteins in the cross-linking family, such as alpha-actinin(α-actinin) and filamin, regularly function as molecular scaffolds that maintain the spatial cross-linking of actin filaments and the stability of the cytoskeletal framework (Rajan et al., 2023a). However, filamin A also functions as a key signaling scaffold, regulating MLC phosphorylation through two independent pathways, namely Rho-associated protein kinase(ROCK) and protein kinase C(PKC), which ultimately modulate cellular contraction (Hong et al., 2024). Myosin II is an actin-binding molecular motor that pulls actin filaments together by sliding along their sides (Rassier and Månsson, 2025). Actin thus achieves effective assembly through the synergistic cooperation of diverse ABPs. In addition to the typical ABPs mentioned above, other ABPs will be discussed in more detail in subsequent chapters on viruses.

### 2.2 Rho GTPases

Among the Rho GTPases, Rac1, Cdc42, and RhoA are the three major classical regulators of the actin cytoskeleton. After being converted into their active GTP-binding forms, they each drive the assembly of different types of actin networks by activating their respective downstream effectors. Rac1 and Cdc42 recruit the Wiskott–Aldrich syndrome protein(WASP) family Verprolin-homologous protein(WAVE) and the WASP, respectively, to activate the Arp2/3 complex, thereby mediating the formation of lamellar and filopodia (Leung and Rosen, 2005; Chen et al., 2017). At the same time, both activate the p21-activated kinase (PAK)–LIM domain kinase (LIMK) cascade, which inhibits the cleavage activity of cofilin through phosphorylation (Edwards et al., 1999). RhoA activates mDia1, a member of the Diaphanous (Dia) subfamily of formins, to promote the linear extension of stress fibers. Formins mediate actin nucleation independent of the Arp2/3 complex (Evangelista et al., 2002). Furthermore, RhoA also activates the ROCK kinase. Activated ROCK enhances myosin II contractile force by inhibiting MYPT1 and stabilizes actin structures via the LIMK-cofilin axis (Kimura et al., 1996; Maekawa et al., 1999). However, these three GTPases do not act independently; rather, there is functional crosstalk among them. Previous studies have demonstrated that Rac1 and RhoA exhibit bidirectional antagonism, forming a bistable system (Byrne et al., 2016). Recent research has revealed that Rac1 activation can also positively activate RhoA. The molecular mediators of this crosstalk are LARG and PRG, which are recruited to the plasma membrane in response to Rac1. LARG responds directly to Rac1 via its PH domain, while PRG is indirectly localized to the cytoplasmic cortex following Rac1-induced actin polymerization through a unique F-actin-binding motif. Together, they convert Rac signaling into Rho activation, consequently driving cell migration (Nanda et al., 2023). The complex regulatory mechanisms of these three enzymes on the actin cytoskeleton will be discussed in subsequent chapters.

## 3 Mechanisms by which RNA viruses hijack actin

RNA viruses have long been major pathogens that jeopardize human public health. Early studies have preliminarily confirmed that the infection process of RNA viruses is highly dependent on the host’s actin cytoskeletal system. During the cellular entry phase, both HIV and IAV rely on the actin cytoskeleton. HIV promotes the aggregation of cell surface receptors with the involvement of actin (Iyengar et al., 1998). IAV relies on dynamic actin and myosin VI to complete endocytosis (Sun and Whittaker, 2007). Treatment with cytochalasin D significantly inhibits the entry of both HIV and IAV into cells. During RSV infection, both monomeric and polymeric forms of actin can activate viral transcription *in vitro*, while filamentous actin participates in viral morphogenesis and budding (Burke et al., 1998). The entry and release processes of DENV both depend on the integrity of the actin cytoskeleton (Wang et al., 2010). Mechanistic studies have revealed that the RSV M protein and the EBOV VP40 protein can colocalize with actin (Han and Harty, 2005; Shahriari et al., 2018). The SARS-CoV-2 spike protein interacts directly with β-actin and γ-actin (Fujimoto et al., 2024). This suggests that RNA viruses have evolved diverse interfaces with the host actin system. In addition, viral ribonucleoprotein complexes (vRNPs) derived from IAV is capable of directed movement along nuclear F-actin (Yu et al., 2022). This directly confirms that actin filaments also function as transport tracks for viral molecules. As mentioned earlier, host cells strictly regulate actin dynamics primarily through the Rho GTPase pathway and downstream actin-binding proteins (ABPs). During their evolution, RNA viruses have gradually learned to hijack various host molecular mechanisms to regulate actin actively, eventually promoting their own infection and transmission. This article summarizes recent advances in the mechanisms by which six important human pathogenic RNA viruses utilize actin (see **Table 1**).

**Table 1:** Overview of the molecular mechanisms by which RNA viruses hijack actin during infection.

Virus name	Functional category	Critical host factor or signaling pathway	Actin dynamics	Biological outcome	Year	Refs.
SARS-COV-2	Viral infection	Formin, Fascin, Arp2/3, Cdc42	Actin assembly	Promoting virus surfing	2021	(Bayati et al., 2021)
		ERM	Actin instability	Cortex softening	2024	(Do et al., 2024)
		PKN-α-actinin	Actin recombination	Promoting virus egress	2023	(Swain et al., 2023)
		NUAK2	Actin stability	Promoting virus entry	2023	(Prasad et al., 2023)
	Pathological effects	TNF-TNFR1-TRADD-TRAF2-RIPK1-MAPK-SDC4-RhoA-ROCK1/2	Actin bundling	Inhibiting cell fusion	2026	(Duan et al., 2026)
		IL-1-IL-1R1-MyD88-IRAK1/4-TRAF6-RhoA-ROCK1/2	Actin bundling	Inhibiting cell fusion	2025	(Zheng et al., 2025)
IAV	Viral infection	Cdc42-N-WASP-Arp2/3	Actin filopodia formation	Promoting virus entry	2022	(Hunziker et al., 2022)
		Cdc42-PAK-CTTN-Arp2/3	Actin filopodia formation	Inhibiting virus entry	2022	(Hunziker et al., 2022)
		FBP17	Actin assembly	Promoting membrane bending	2022	(Tang et al., 2022)
		Claudin-11	Actin depolymerization	Promoting CCP maturity	2025	(Yu et al., 2025)
		ADD1-RhoA-MLC	Actin recombination	Promoting virus uncoating	2025	(Jiang et al., 2025)
		ARHGAP1	Actin stability	Promoting virus budding	2022	(Kuroki et al., 2022)
RSV	Viral infection	PI3K-AKT-RhoA-Cofilin	Actin recombination	Promoting membrane fusion	2020	(Chen et al., 2020)
		Palladin	Actin associated scaffold	Promoting virus egress	2024	(Shahriari and Ghildyal, 2024)
	Pathogenesis​ effects	PI3K-Cdc42-Cofilin	Actin polymerization	Promoting cell fusion	2022	(Chen et al., 2022)
		CCTN-Rap1-Rac1	Actin depolymerization	Disrupting epithelial barrier	2022	(Gao et al., 2022)
DENV	Viral infection	C-ABL	Actin recombination	Promoting virus entry	2025	(Carreño-Flórez et al., 2025)
		DUSP5-ERK-MLCK-MLC2-Myosin IIB	Actin recombination	Inhibiting virus entry	2023	(Liang et al., 2023)
		CapZ	unknown	Promoting virus releasing from endosome	2024	(Zhu et al., 2024)
EBOV	Viral infection	FLNa	Actin cross-linking	Promoting macropinocytosis	2023	(Shepley-McTaggart et al., 2023)
		FLNb	Actin cross-linking	Inhibiting macropinocytosis	2023	(Shepley-McTaggart et al., 2023)
		Rac1-Wave1-Arp2/3	Actin tail formation	Promoting virus transport	2020	(Grikscheit et al., 2020)
		CAPG	Actin stability	Promoting virus egress	2022	(Mori et al., 2022)
HIV	Viral infection	Gp120-CXCR4-Rac1-PAK-LIMK-cofiln	Cortical actin stability	Promoting virus entry	2011	(Vorster et al., 2011)
		Gp120-CXCR4/CCR5-WAV2-Arp2/3	Actin branching formation	Promoting PIC transport	2014	(Spear et al., 2014)
		PSP-IRE1α-PKR-Cofilin1	Actin polymerization	Inhibiting virus entry	2023	(Alvarez-Rivera et al., 2023)
		Arf1	unknown	Promoting virus gag transport	2025	(Smith et al., 2025)
		Arf6	unknown	Stabilizing virus gag	2025	(Smith et al., 2025)
		Arpin-Arp2/3	Actin nucleation inhibition	Promoting virus assembly	2023	(Dibsy et al., 2023)
		Rab35-MICAL1	Actin depolymerization	Promoting virus budding	2024	(Serrano et al., 2024)
	Pathogenesis​ effects	Nef-CK2-WAVE2-Arp2/3	Lamellipodia abnormality	Immune dysfunction	2026	(Dunn et al., 2026)
		Nef-PAK2-Cofilin1	Actin stability	Inhibiting IFN response	2025	(Laliberté et al., 2025)
		Cofilin	Actin depolymerization	Disrupting the function of CD4+ T cells(clinical)	2019	(He et al., 2019)
RV	Viral infection	VP4	Cortical actin recombination, stress fiber decrease	Promoting viroplasm assembly	2022	(Vetter et al., 2022)
CHIKV	Viral infection	MK2/MK3-LIMK1-Cofilin	Actin filopodia formation	Promoting virus egress	2021	(Mamidi et al., 2021)
		Rac1-PAK1-Arp2	Actin filopodia formation	Promoting virus replication	2024	(Aïqui-Reboul-Paviet et al., 2024)
NiV	Viral infection	Arp2/3	Actin branch formation	Promoting virus budding	2025	(Wang et al., 2025)
DBV	Viral infection	NSs	Actin recombination	Inhibiting virus entry	2022	(Liu et al., 2022)
HTNV	Viral infection	GBP1	unknown	Inhibiting CME	2024	(Gu et al., 2024)
MRV	Viral infection	ANXA2	Actin stability	Inhibiting VF formation	2026	(Tenorio et al., 2025)

See the list of abbreviations at the end of the paper for the full forms of all abbreviations.

### 3.1 SARS-CoV-2

SARS-CoV-2 is a coronavirus and the primary pathogen responsible for the COVID-19 pandemic (Huang et al., 2020). Previous studies have shown that the dynamic assembly of actin interferes with the endocytosis of coronaviruses (Owczarek et al., 2018). Filopodia are protrusions of the plasma membrane composed of actin (Miki et al., 1998). Single-virus tracking analyses in recent work have demonstrated that SARS-CoV-2 single-virus-like particles exploit filopodia to travel to entry sites through two different mechanisms, “surfing” and “grabbing”, which circumvents random diffusion across the plasma membrane. Furthermore, the formation of filopodia depends on Cdc42 activity and on actin-associated proteins such as fascin, formin, and Arp2/3 (Zhang et al., 2023). The preferred entry mechanism for SARS-CoV-2 is via the Receptor Binding Domain (RBD) of the Spike protein binding to the Angiotensin-Converting Enzyme 2 (ACE2) receptor, followed by laminin-mediated endocytosis (Bayati et al., 2021). A study using atomic force microscopy demonstrated that the interaction between the RBD of the SARS-CoV-2 Spike protein and ACE2 leads to a decrease in cytoplasmic cortical stiffness. The specific mechanism may involve this interaction inducing the dephosphorylation of the Ezrin/Radixin/Moesin (ERM) proteins (Do et al., 2024). ERM proteins are actin-membrane linkers that connect cortical actin to the plasma membrane and promote Spike protein assembly (Wang et al., 2026). Upon dephosphorylation, ERM proteins become inactivated and lose their ability to act as linkers, leading to a decrease in cortical stiffness (Faure et al., 2004; Kihara et al., 2021). Furthermore, cofilin-1, an actin-severing protein, is downregulated during viral endocytosis; this change promotes accumulation of polymerized actin (Yeung-Luk et al., 2023). This suggests that ERM and cofilin may jointly coordinate actin cytoskeletal reorganization during SARS-CoV-2 entry. As mentioned earlier, α-actinin is a key actin-binding protein. When SARS-CoV-2 infects lung cells, α-actinin, under the regulation of Protein Kinase N (PKN), participates in significant F-actin reorganization, consequently promoting the late-stage release of SARS-CoV-2 (Swain et al., 2023). NUAK family kinase 2 (NUAK2) is a serine/threonine protein kinase that regulates cytoskeletal remodeling (Vallenius et al., 2011). Studies have shown that NUAK2 is a novel host-dependent factor for SARS-CoV-2 entry. SARS-CoV-2 infection leads to endoplasmic reticulum(ER) stress and the Unfolded Protein Response (UPR), which is transmitted to uninfected bystander cells. At this point, NUAK2 transcription levels in the uninfected cells are significantly elevated, which, in turn, increases surface ACE2 levels and stabilizes actin, ultimately assisting viral entry (Prasad et al., 2023).

In addition to facilitating viral entry and release, related actin-associated host factors additionally play a role in the spread of the virus to neighboring cells. A distinctive feature of SARS-CoV-2-infected cells is the formation of multinucleated syncytia resulting from intercellular fusion, which facilitates accelerated intercellular transmission of the virus (Zhu et al., 2020). Recent studies have found that branching actin polymerization induced by activation of the Rac-Arp2/3 pathway is essential for virus-induced cell fusion (Kou et al., 2025). In contrast, RhoA-mediated actin polymerization inhibits cell fusion. Tumor necrosis factor (TNF) is an important pro-inflammatory cytokine that typically inhibits viral infection. At the same time, it also regulates actin polymerization (Wong and Goeddel, 1986; Goldblum et al., 1993). Duan et al. (2026) found that TNF can induce the formation of actin bundles at cell-cell junctions to inhibit SARS-CoV-2-induced cell fusion. The TNF/Tumor Necrosis Factor Receptor(1TNFR1)-mediated Mitogen-Activated Protein Kinase (MAPK) signaling pathway transmits signals to Syndecan-4 (SDC4), which, in turn, requires activation via the RhoA/ROCK pathway to promote actin filament formation ultimately. Interleukin-1 beta (IL-1β) is another important pro-inflammatory cytokine (Dinarello et al., 1974). Recent studies have found that it can also inhibit SARS-CoV-2-induced cell fusion. IL-1β signals through an atypical IL-1 receptor-dependent pathway and ultimately activates the RhoA/ROCK pathway, driving the enrichment of actin bundles between cells (Zheng et al., 2025). This illustrates the unprecedented antiviral functions of cytokines during viral infection. **Figure 2** illustrates the double role of actin in the SARS-CoV-2 infection process.

**Figure 2:**
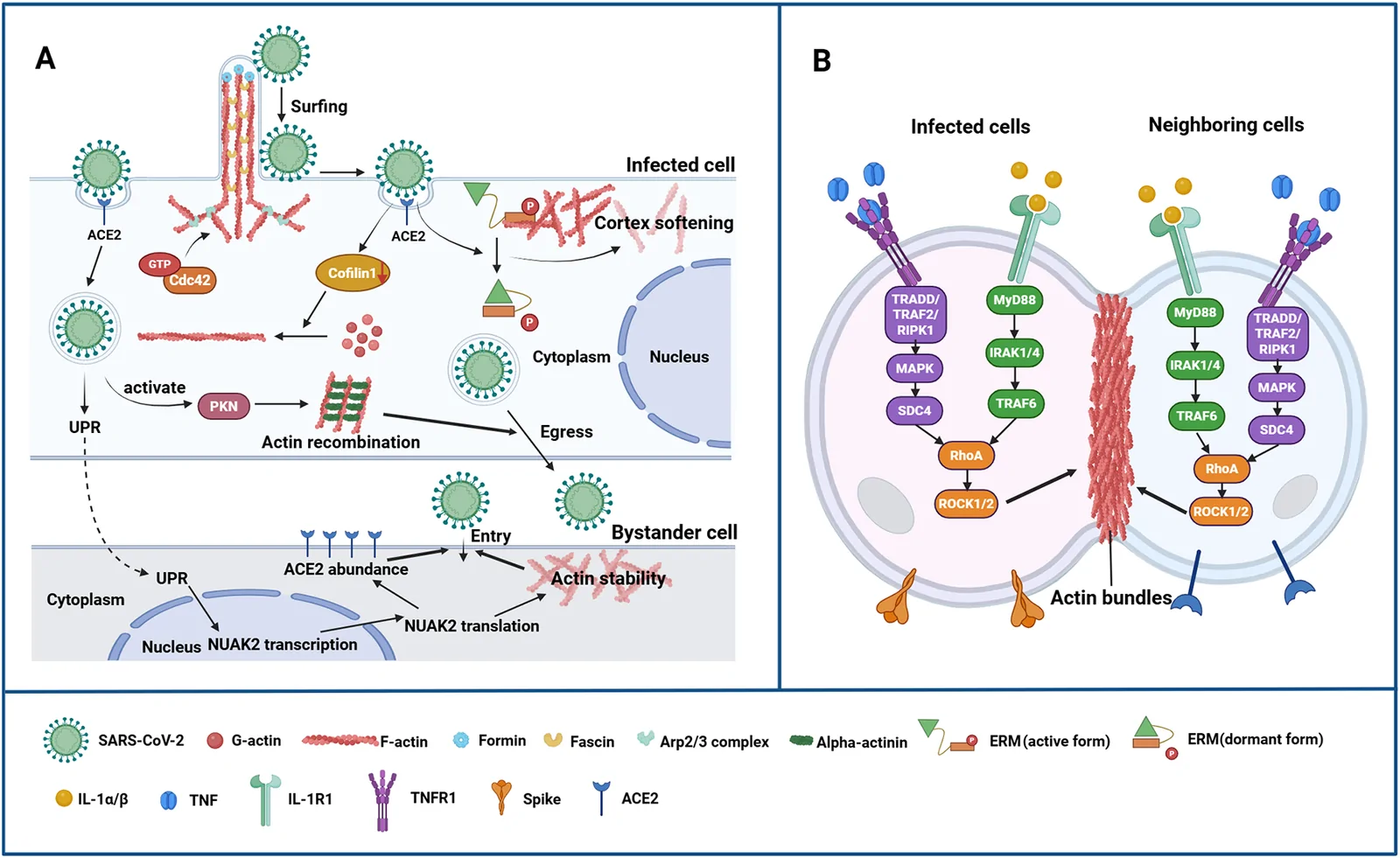
Dual regulation of the actin cytoskeleton by SARS-CoV-2 infection. Black bold arrows indicate promotion. **(A)** Molecular mechanisms by which SARS-CoV-2 hijacks actin during infection. During viral entry, Formin, Fascin, the Arp2/3 complex, and active Cdc42 mediate filopodia formation. Following viral binding to ACE2, ERM undergoes dephosphorylation and cofilin-1 is downregulated, leading to cortical softening and increased actin polymerization. In the late stages of viral infection, activated PKN further regulates α-actinin to reorganize the actin cytoskeleton, eventually promoting viral release. The UPR response induced by viral infection, when transmitted to bystander cells, promotes the expression of NUAK2, causing the accumulation of ACE2 and the stabilization of actin, which ultimately facilitates viral entry. **(B)** Actin inhibits cell fusion. Both TNF and IL-1 rely on the RhoA/ROCK pathway to induce the formation of intercellular actin bundles, thereby inhibiting cell fusion. See the list of abbreviations at the end of the paper for the full forms of all abbreviations. Created in BioRender. Jie, G. (2026) https://BioRender.com/hqt6qss.

### 3.2 IAV

IAV is a zoonotic pathogen that causes respiratory infections in humans and is prone to causing seasonal influenza and pandemics worldwide (Feng et al., 2022; Hagiya et al., 2023). Both the entry and transport of IAV require actin. Filopodia are finger-like projections on the cell surface with bundles of actin filaments serving as their core scaffold. Many viruses are typically able to slide along filopodia to enter cells (Lehmann et al., 2005). IAV relies on cellular filopodia to “surf” to the entry site, but it can actively induce filopodia formation to facilitate its entry into the cell further. IAV infection induces Cdc42-dependent activation of N-WASP, which subsequently localizes to the cell cortex and enhances filopodia formation. IAV can hijack these filopodia to enhance efficient endocytosis-mediated entry (Martinez-Quiles et al., 2001). However, host cells limit this virus-induced filopodia formation because Cdc42 activation also activates cortactin (CTTN) via type I PAK. CCTN is an ABP that localizes to the actin cortex (Wu and Parsons, 1993). Activated CTTN migrates to the cell cortex and displaces N-WASP at actin filament bifurcations, thereby preventing filopodia formation and limiting IAV uptake (Hunziker et al., 2022). This suggests that host cells can suppress filopodia formation by activating signaling pathways distinct from those triggered by viral infection. Formin-binding protein 17 (FBP17) is a key regulator of membrane invagination that further prolongs membrane deformation by enhancing cdc42-, WASP/N-WASP-, and Arp2/3-dependent actin nucleation and polymerization (Takano et al., 2008). A study found that FBP17 also mediates membrane bending during the IAV entry phase, which facilitates viral internalization. Prior to this, the clathrin-coated structure (CCS) of IAV first recruits a sphingomyelin (SM)-cholesterol complex nanodomain. This nanodomain is essential for recruiting the membrane-bending protein FBP17. FBP17 activates local actin assembly, thereby inducing contraction of the IAV-containing CCS neck (Tang et al., 2022). Claudin-11 is a four-transmembrane protein encoded by the claudin-11 gene and is a member of the claudin family, which forms tight junctions in the cell membrane (Bronstein et al., 1996; Morita et al., 1999). Studies have shown that claudin-11 also plays an essential role in IAV clathrin-mediated endocytosis. During viral internalization, claudin-11 directly interacts with the metabotropic glutamate receptor 2 (mGluR2) and breaks the interaction between mGluR2 and KCa1.1. mGluR2 is the receptor that triggers clathrin-mediated endocytosis. Together with mGluR2, claudin-11 is internalized along with the influenza virus, thereby regulating F-actin depolymerization and promoting the maturation of clathrin-coated pits (CCPs) (Yu et al., 2025). The specific mechanism by which claudin-11 induces F-actin depolymerization remains to be studied, but based on related studies, it may be associated with inactivation of the RhoA signaling pathway (Li et al., 2019). Adducin-1 (ADD1) is an actin-capping protein that promotes the cross-linking of actin filaments with spectrin and stabilizes the length of filamentous actin (Li et al., 1998). ADD1 has been identified as a key host factor for IAV replication (Zou et al., 2025). ADD1 promotes the fusion of virus-containing vesicles with early endosomes by coordinating actin reorganization. At the same time, the virus detaches from actin filaments and moves rapidly along microtubules. Once the viral envelope fuses with the endosomal membrane, ADD1 promotes viral uncoating by regulating the RhoA/MLC pathway (Jiang et al., 2025). The temporal and spatial dynamics of IAV gene transport within the cell nucleus have been a research gap in recent years. Using single-particle tracking techniques, Yu et al. (2022) discovered a nuclear F-actin-dependent pathway for the transport of IAV vRNPs from the nucleus to the cytoplasm. IAV infection significantly induces the formation of F-actin microfilaments within the cell nucleus. Concurrently, vRNPs undergo slow, directed movement along nuclear F-actin, similar to that of IAVs along cytoplasmic microfilaments. This reveals a new target for antiviral therapy. Rho GTPase-activating protein 1 (ARHGAP1) is a newly identified GTPase-activating protein (GAP) of the Rho GTPase family that colocalizes with Rab11a recycling endosomes (Res) in IAV-infected cells. Rab11a-positive recycling endosomes (Rab11a REs) frequently act as carriers for the transport of influenza virus vRNP from the perinuclear endosomal recycling compartment to the plasma membrane (Momose et al., 2011). During IAV endocytosis, ARHGAP1 is transported alongside Rab11a-mediated vRNP transport. During this process, actin filaments are stabilized to promote the assembly of hemagglutinin (HA) molecules at the plasma membrane, ultimately forming the bud zone. This ensures the viral surface integrity necessary for viral survival, therewith promoting viral stability and immune escape (Kuroki et al., 2022). The molecular mechanism by which IAV hijacks actin is shown in **Figure 3**.

**Figure 3:**
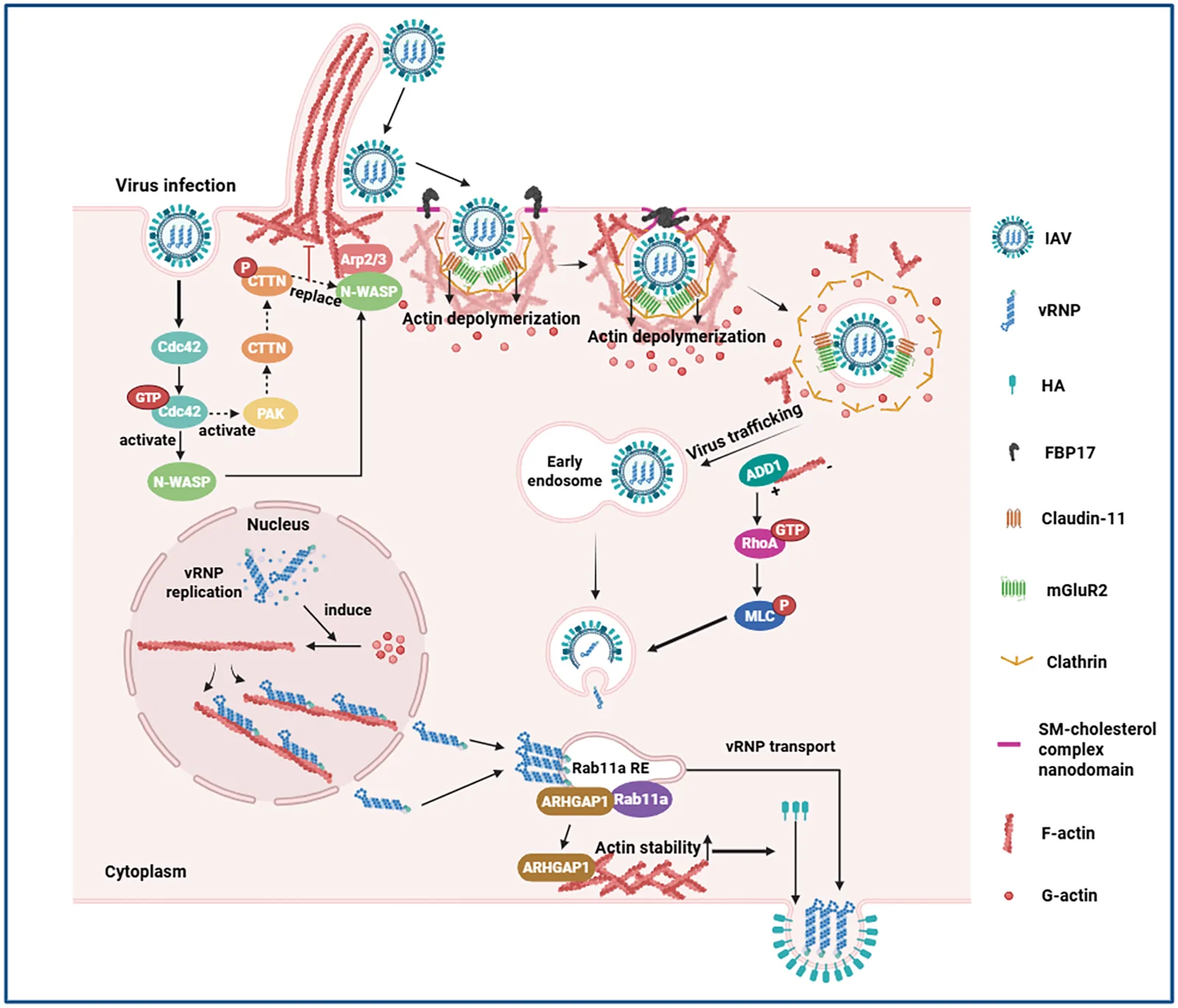
Molecular mechanisms by which IAV hijacks actin during infection. Black bold arrows indicate promotion. Early viral infection activates the Cdc42/N-WASP/Arp2/3 pathway, promoting the formation of filopodia to facilitate viral entry. Concurrently, the PAK/CTTN pathway is also activated; CTTN competitively binds to the Arp2/3 complex with N-WASP, exerting an opposing effect. During virus-mediated clathrin-mediated endocytosis, FBP17 promotes membrane invagination through facilitating localized actin assembly, while Claudin-11 promotes actin depolymerization to drive CCP maturation; together, they facilitate viral entry. During the fusion of the virus with early endosomes, ADD1 promotes fusion by cross-linking actin. Subsequently, ADD1 also promotes viral uncoating by activating the RhoA/MLC pathway. The assembly of viral vRNP in the cell nucleus induces actin polymerization to form microfilaments, which in turn assist vRNP in exiting the nucleus. During the transport of vRNP to the plasma membrane via the Rab11a receptor endosome (RE), ARHGAP1—which is transported alongside vRNP—promotes the aggregation of HA on the plasma membrane to form a budding site by mediating actin stabilization. See the list of abbreviations at the end of the paper for the full forms of all abbreviations. Created in BioRender. Jie, G. (2026) https://BioRender.com/bx0v5hc.

### 3.3 RSV

RSV is one of the leading causes of lower respiratory tract infection morbidity and fatality worldwide, with the highest burden among children and the elderly (Chen et al., 2025). PI3K is a family of cellular kinases that typically acts as an upstream signal for actin reorganization (Wennström et al., 1994). PI3K has been shown to promote RSV entry (Krzyzaniak et al., 2013). Chen et al. (2020) further explained the specific molecular mechanisms by which PI3K regulates RSV entry and identified relevant inhibitors. Upon activation of the PI3K signaling pathway by RSV, the downstream molecules RhoA and cofilin are upregulated, therewith promoting actin cytoskeletal reorganization and the fusion of RSV with the cell membrane during the entry phase. In contrast, the potent PI3K inhibitor PIK-24 significantly reversed this process. The RSV matrix protein (M) has been shown to bind directly to F-actin *in vitro*. Upon disruption of the microtubules, M diffuses from the main cytoplasm to the space between the cytoplasm and the nucleus. Furthermore, knockout experiments have demonstrated that both the N-terminus and C-terminus of M are involved in its interaction with actin (Mitra et al., 2012; Shahriari et al., 2018). Palladin is an actin-binding protein that can directly bind to and cross-link F-actin (Dixon et al., 2008). Recent studies have shown that palladin may promote RSV budding and release by mediating M-actin interactions. Immunoprecipitation has confirmed that palladin also interacts with M. Furthermore, in RSV-infected cells, after removal of nuclear and cytoplasmic proteins, complexes containing M were observed to form elongated aggregates. This suggests that palladin can mediate the movement of M and microtubules toward the cell surface, finally leading to viral release (Shahriari and Ghildyal, 2024). However, the basic mechanisms call for more investigation. Actin is also involved in the membrane fusion stage following RSV infection. This is mainly achieved by inducing membrane fusion through increased actin polymerization via the PI3K/Cdc42/Cofilin signaling axis. PIK-24 inhibits membrane fusion by blocking PI3K signaling activation through its targeting of the PI3K p85α regulatory subunit (PI3K p85α) protein (Chen et al., 2022). This constitutes a prospective lead compound for the development of novel anti-RSV drugs. Breaching the epithelial barrier is a critical step in the viral infection process. RSV infection increases airway epithelial barrier permeability and disrupts apical junctional complexes (AJCs) in both *in vivo* and *in vitro* models (Rezaee et al., 2017; Smallcombe et al., 2019). A study has delineated the molecular mechanisms involved. In RSV-infected epithelial cells, CCTN protein levels are reduced. This attenuates the activity of the downstream Ras-related protein 1 (Rap1)/Rac1 signaling pathway, leading to F-actin depolymerization. The instability of the F-actin network ultimately results in the disassembly of AJCs and increased permeability of the epithelial barrier (Gao et al., 2022). **Figure 4** shows the role of actin in promoting RSV infection.

**Figure 4:**
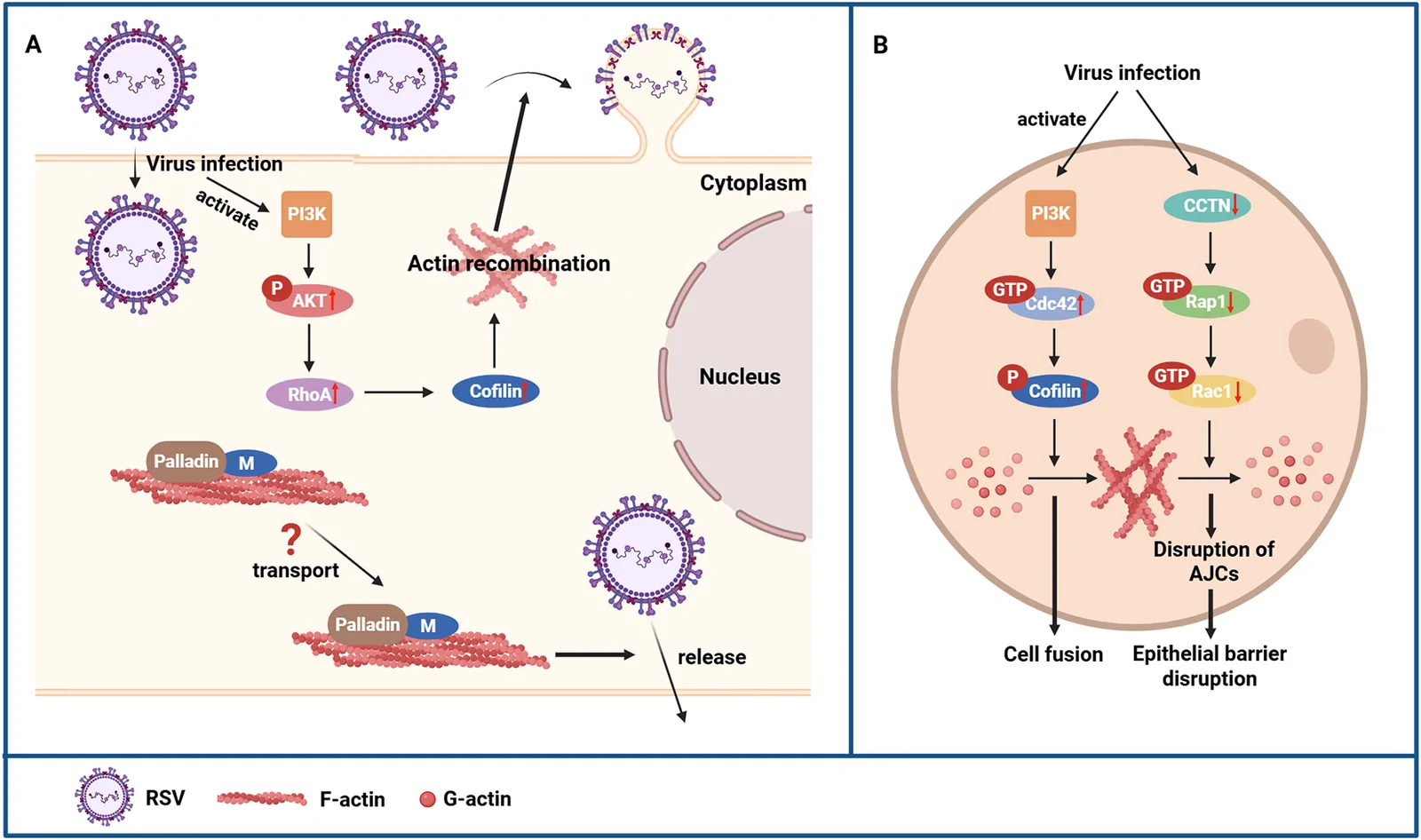
RSV exploitation of the actin regulatory network. Black bold arrows indicate promotion. The red question mark indicates that this is pending verification. **(A)** The molecular mechanism by which RSV hijacks actin during infection. Early viral infection activates the PI3K pathway, leading to actin reorganization and eventually promoting membrane fusion. The movement of the viral M protein toward the plasma membrane depends on palladin and actin, therewith facilitating viral release. **(B)** Pathogenic effects resulting from RSV-mediated actin remodeling. Following viral infection, the Cdc42/cofilin pathway is activated by PI3K, promoting actin polymerization and eventually facilitating cell fusion. Additionally, downregulation of CCTN inhibits Rac1 activation, promotes actin depolymerization, and, consequently, damages the AJC and increases epithelial barrier permeability. See the list of abbreviations at the end of the paper for full definitions of all abbreviations. Created in BioRender. Jie, G. (2026) https://BioRender.com/qwrx7yy.

### 3.4 DENV

DENV is a common mosquito-borne virus that causes approximately 100 million symptomatic cases and 10,000 deaths annually (Messina et al., 2019). Previous studies have demonstrated direct interactions between DENV proteins and actin, suggesting that the virus relies on actin to facilitate infection (Jitoboam et al., 2016). Furthermore, during the DENV release phase, the microfilament motor myosin VC plays a key role, further underlining the importance of actin in DENV infection (Xu et al., 2009). Abelson tyrosine kinase(c-ABL) is a non-receptor tyrosine kinase that regulates multiple signaling pathways, including cell proliferation and cytoskeletal remodeling. Additionally, c-ABL contains an actin-binding domain at its C-terminus, constituting it an important regulator of actin dynamics (Van Etten et al., 1994). When DENV-2 infects Vero cells, c-ABL enhances viral entry and subsequent accumulation of the envelope protein (ENV) by inducing actin rearrangement. Moreover, imatinib, a specific c-ABL inhibitor, attenuates DENV-induced F-actin reorganization (Carreño-Flórez et al., 2025). Dual specificity phosphatase 5 (DUSP5) is a cellular phosphatase that inactivates the MAPK family by dephosphorylating phosphoserine and phosphotyrosine residues (Ishibashi et al., 1994). Liang et al. (2023) found that DUSP5 potently inhibits DENV replication. The specific mechanism entails DUSP5 negatively regulating the Extracellular Signal-Regulated Kinase (ERK)-MLCK-myosin IIB signaling axis to inhibit F-actin reorganization, thereby limiting viral entry into host cells. This provides a powerfully broad-spectrum antiviral target. Actin capping protein (CapZ) is a capping protein for actin in the cytoskeleton that prevents filament assembly by capping the plus ends of actin filaments (Casella et al., 1986). In addition, CapZ can regulate endosomal transport by modulating the density of F-actin around endosomal vesicles and recruiting Rabaptin-5 and Rabex-5 (Wang et al., 2021). Rabaptin-5 and Rabex-5 are, respectively, the effector protein and the guanine exchange factor of the RAB5 small GTPase (Horiuchi et al., 1997). Another recent study has found that the regulation of endosomes by CapZ is time-dependent. When flaviviruses such as DENV form early endosomes (EEs), CapZ localizes to the EEs and recognizes the prominent phosphamide Phosphatidylinositol 3-phosphate (PI3P) on their surface. Subsequently, after the fusion of two EEs, CapZ is released from the mature EE; at this point, PI3P is converted to phosphatidylinositol 3,5-bisphosphate (PI (Liao et al., 2018; Bibeau et al., 2020)P^2^), and the Ras-related protein Rab-5A (RAB5) is replaced by the Ras-related protein Rab-7a (RAB7). At this point, the release of the viral genome from endosomal vesicles increases (Zhu et al., 2024). However, how CapZ regulates actin during this process remains to be further investigated. The effect of actin on the early infection stage of DENV is shown in **Figure 5**.

**Figure 5:**
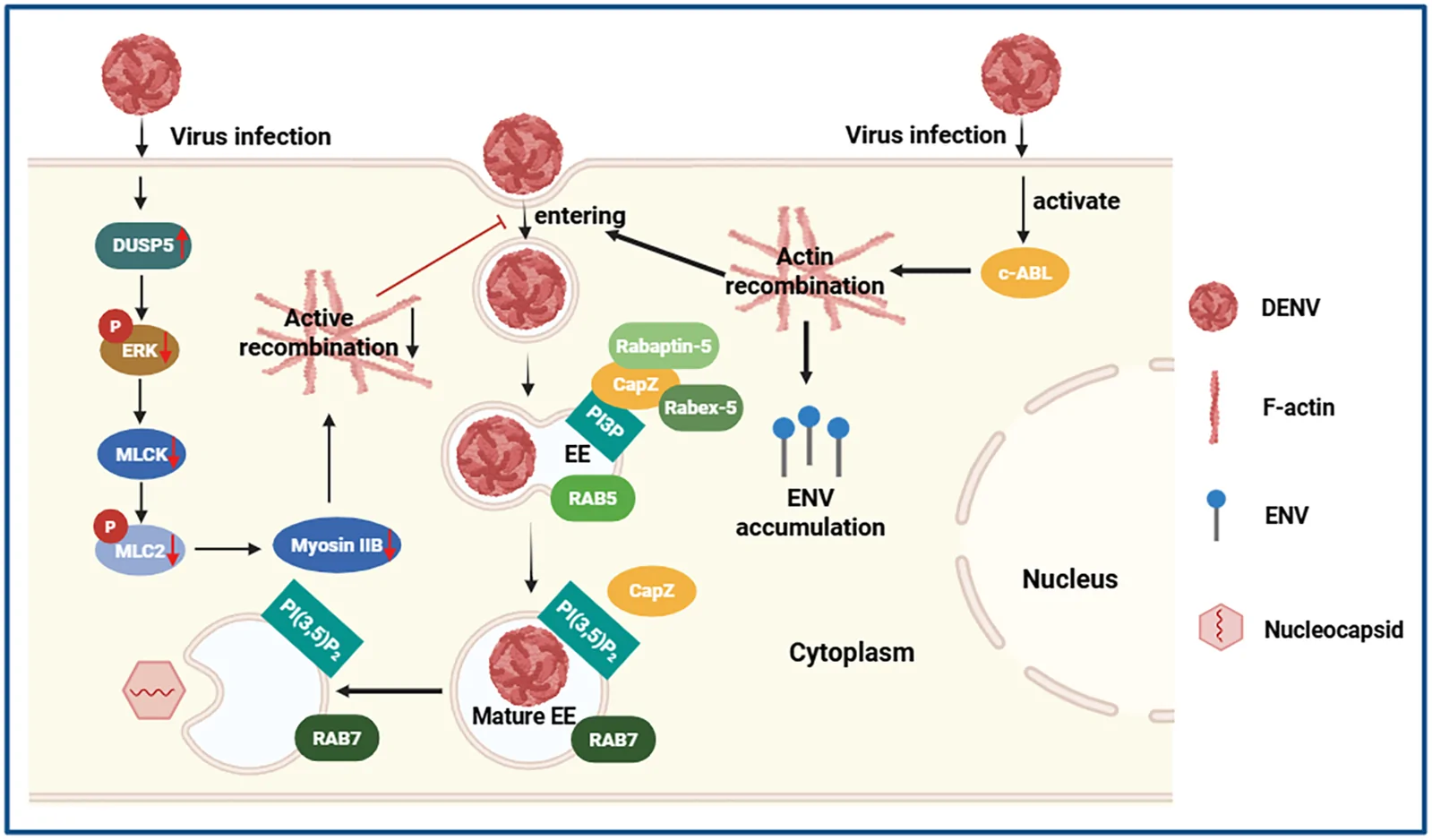
Dual regulation of actin during early DENV infection. Black bold arrows indicate promotion. Early viral infection activates c-ABL to regulate actin reorganization, consequently promoting ENV accumulation and viral entry. At the same time, upregulation of DUSP5 inhibits the ERK-MLCK-myosin IIB signaling axis, limiting actin reorganization and, in turn, inhibiting viral entry. During the EE formation stage, CapZ binds to PI3P on the EE and recruits Rabaptin-5 and Rabex-5. Upon EE fusion, CapZ departs from the endosome, and newly formed PI (3,5)P^2^ and RAB7 promote the release of the viral nucleocapsid from the endosome. See the list of abbreviations at the end of the paper for the full forms of all abbreviations. Created in BioRender. Jie, G. (2026) https://BioRender.com/8xp44ds.

### 3.5 EBOV

EBOV is a highly contagious filovirus with a global average case fatality rate exceeding 50% (Izudi and Bajunirwe, 2024). Filamin is an actin-binding protein consisting of three subtypes: Filamin A (FLNa), Filamin B (FLNb), and Filamin C (FLNc). Studies have shown that filamin also serves an important function in the filovirus life cycle. Specifically, the presence of FLNa promotes EBOV entry into cells, whereas FLNb inhibits viral entry. Further experimental observations indicate that the effects of FLNa and FLNb on EBOV entry may be mediated by the regulation of macropinocytosis (Shepley-McTaggart et al., 2023). Macropinocytosis mediates the endocytosis of filoviruses and is highly dependent on actin (Nanbo et al., 2010; Saeed et al., 2010). Similar to DNA viruses such as vaccinia virus, the long-distance intracellular transport of EBOV also relies on actin comet tails. Furthermore, this transport process is closely associated with the Rac1-Wave1-Arp2/3 pathway (Grikscheit et al., 2020; Dolnik and Becker, 2022). Previous studies have shown that actin can be packaged into EBOV-like particles and co-localizes with VP40 (Han and Harty, 2005). VP40 is a matrix protein of EBOV that induces viral particle budding. When VP40 initiates budding, viral glycoproteins(GPs) are recruited to the budding site to promote envelope formation (Noda et al., 2002). Subsequent studies using cryo-electron tomography further confirmed the interaction between VP40 and actin filaments, which may be one of the mechanisms promoting viral budding (Wan et al., 2020; Winter and Chlanda, 2021). Macrophage Capping Protein (CAPG) is an actin-binding protein that promotes the stability of actin filaments to prevent their further elongation (Yu et al., 1990). During EBOV infection, CAPG promotes viral budding, and CAPG and VP40 colocalize along actin filaments. Proximity-linking and cleavage of green fluorescent protein (GFP) recombinants unveiled a strong interaction between CAPG and VP40, mediated by the S1 domain of CAPG. This suggests that CAPG links actin filament stabilization to viral release from cells, thereby regulating viral escape from infected cells (Mori et al., 2022). EBOV utilization of actin is shown in **Figure 6**.

**Figure 6:**
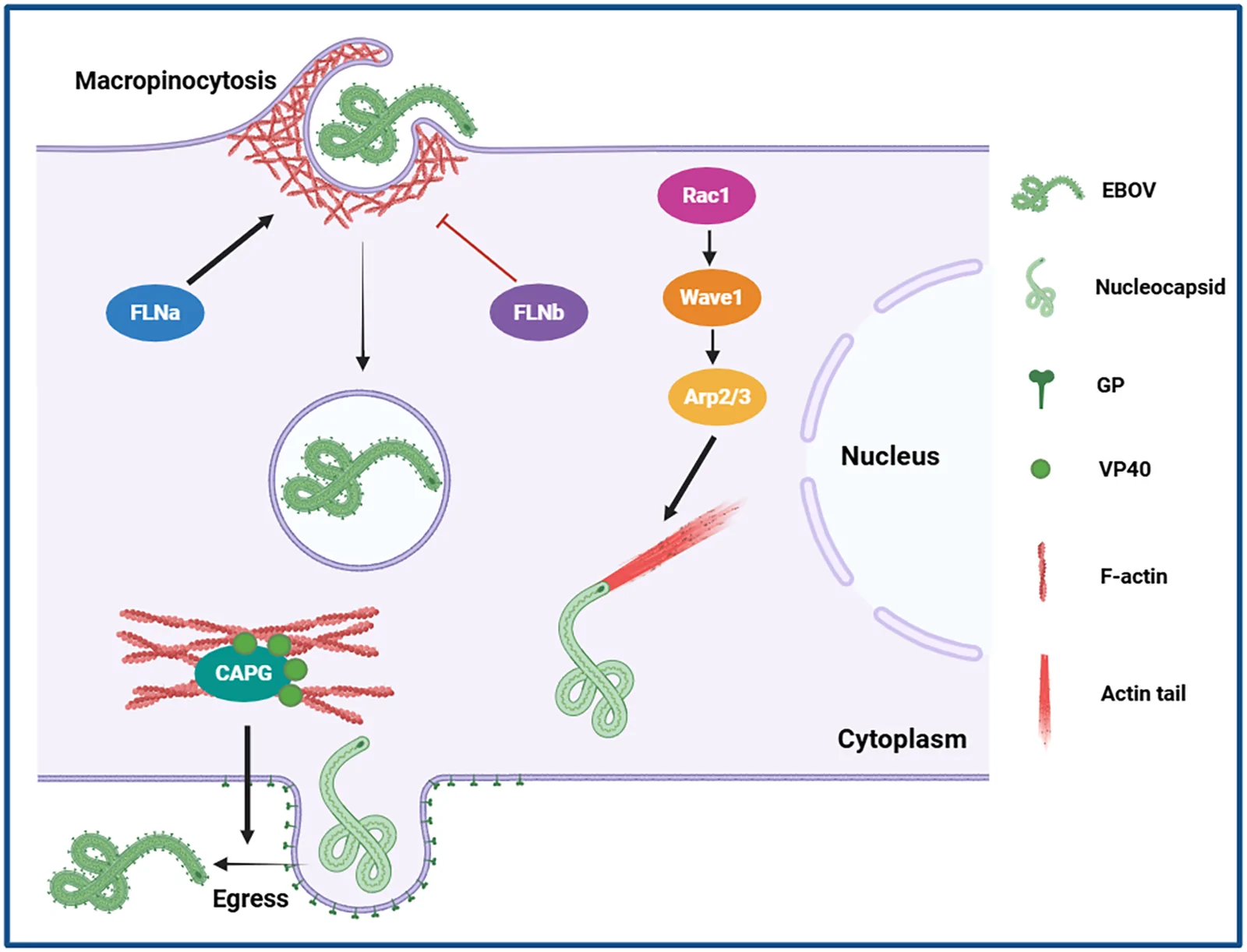
Molecular mechanism of EBOV hijacking of actin. Black bold arrows indicate promotion. During viral entry, FLNa and FLNb modulate viral entry by promoting and inhibiting viral macropinocytosis, respectively. The macropinocytosis process is actin-dependent. Intracellular transport of the virus requires an actin comet tail mediated by the Rac1-Wave1-Arp2/3 pathway. During late stages of viral infection, CAPG and VP40 colocalize along actin filaments, promoting viral release. See the list of abbreviations at the end of the paper for the full forms of all abbreviations. Created in BioRender. Jie, G. (2026) https://BioRender.com/e1bzr34.

### 3.6 HIV

HIV is a retrovirus that causes acquired immunodeficiency syndrome(AIDS) and stays a major global public health threat (Hu et al., 2023; Cheng et al., 2025). The persistence of viral reservoirs is a major obstacle to curing HIV infection, and quiescent CD4+ T cells are considered one of the primary viral reservoirs (Siliciano et al., 2003). Earlier studies have already identified an interaction between HIV and actin, which provides a basis for further research (Liu et al., 1999). During HIV infection, chemokine receptors have been shown to be closely associated with actin dynamics. Upon binding to CXCR4, the HIV envelope protein gp120 induces the activation of cofilin, which in turn lifts the cortical actin constraint on resting CD4 T cells. This facilitates viral entry (Yoder et al., 2008). In addition, the binding of gp120 to CXCR4 transiently activates LIMK1 via the Rac1-PAK-LIMK1 pathway. At this point, the inactivation of cofilin stabilizes the cortical filamentous actin, reduces CXCR4 endocytosis, and thereby facilitates the entry of the virus (Vorster et al., 2011; Yi et al., 2017). This demonstrates the flexibility with which HIV uses CXCR4 signaling to regulate actin. In addition to utilizing cofilin, HIV can also activate the WAVE2-Arp2/3 pathway via CXCR4 and CCR5, thereby promoting actin branching (Spear et al., 2014). This is conducive to the transport of the viral preintegration complex (PIC) to the nucleus, thereby accelerating the subsequent gene integration. Meanwhile, Cameron et al (Cameron et al., 2010). demonstrated that the three chemokine receptors CCR7, CXCR3, and CCR6 can respectively bind to the chemokines CCL19/CCL21, CXCL9/CXCL10, and CCL20. This leads to the dephosphorylation of cofilin and the rapid rearrangement of actin, ultimately promoting the establishment of the latency period of HIV-1. CXCL10 is also known as interferon-γ-induced protein 10 (IP-10). Recently, Wang et al (Wang et al., 2021). further demonstrated that IP-10/CXCR3 reshapes actin via the LIMK-cofilin pathway, thereby promoting HIV entry into cells and DNA integration. The formation of the HIV latent reservoir in terminally quiescent CD4+ T cells was promoted. Prior to this, IP-10 levels in the plasma of HIV-infected individuals had also increased significantly and were positively correlated with HIV viral load and the size of the viral reservoir. This suggests that IP-10 is an important target for suppressing latent HIV infection. Previous studies have demonstrated that polysaccharopeptide (PSP), a bioactive extract from Coriolus versicolor, exhibits anti-HIV-replication properties in monocytes (Collins and Ng, 1997; Rodríguez-Valentín et al., 2018). Subsequent studies found that PSP can target actin to inhibit the virus. Following PSP treatment of monocytes, the UPR marker inositol-requiring protein 1 alpha (IRE1α) induces the phosphorylation of the interferon-induced double-stranded RNA-dependent protein kinase (PKR), which in turn leads to the upregulation of cofilin-1 phosphorylation. This series of reactions reverses HIV-induced actin remodeling during the course of infection, thereby limiting early viral entry (Alvarez-Rivera et al., 2023). ADP-ribosylation factor (Arf) proteins are small GTPases that are essential for the transport of HIV-1 Gag to the plasma membrane (Joshi et al., 2008). PI (Taylor et al., 2011; Liao et al., 2018)P_2_ on the plasma membrane is the determining factor for Gag plasma membrane targeting (Ono et al., 2004). New research reveals that Arf1 and Arf6 work together to transport HIV-1 Gag to the plasma membrane. In this process, the dynamic GTP/GDP cycle of Arf1 makes certain that Gag is delivered in the correct direction to phosphatidylinositol 4,5-bisphosphate (PI (Taylor et al., 2011; Liao et al., 2018)P^2^)-rich regions of the plasma membrane. Meanwhile, the dynamic GTP/GDP cycle of Arf6 prevents the PI (Taylor et al., 2011; Liao et al., 2018)P^2^ microdomains and Gag from being internalized into endosomes. Through their coordinated action, the virus can complete assembly and release successfully (Smith et al., 2025). However, the link between Arf1, Arf6, and actin during this process remains to be verified. Arpin is a host factor that inhibits the Arp2/3 complex and assists in cell migration (Fregoso et al., 2022). Recent studies have revealed that Arpin also serves an essential function in promoting HIV-1 assembly and release. When HIV-1 infects T cells, the Gag protein, which mediates virus assembly, reaches the plasma membrane and assembles inside regions of the cytoplasmic membrane where cortical F-actin density is lower. Gag can then recruit Arpin to enable this process, as a reduction in actin branching promotes the assembly of HIV-1 Gag (Dibsy et al., 2023). A molecule interacting with CasL-1 (MICAL1) belongs to a conserved flavin-containing oxidoreductase family capable of locally depolymerizing actin filaments by oxidizing specific methionine residues in actin filaments (Rajan et al., 2023b). Researchers have also found that MICAL1-mediated cortical actin depolymerization promotes HIV budding and release. During this process, the MICAL1 activator, the Ras-related protein Rab-35 (Rab35), is also recruited to the budding site. Upon MICAL1 depletion, F-actin abnormally accumulates at the viral budding site, causing the accumulation of incompletely budded viruses on the plasma membrane and the inhibition of viral release (Serrano et al., 2024).

In addition to influencing the HIV infection process, actin can also assist the virus in promoting pathological outcomes. Previous studies have shown that the HIV protein Nef can interact with actin (Fackler et al., 1997). Recent research has revealed that HIV Nef can regulate actin by inhibiting the WAVE2-ARP2/3 pathway. When HIV infects CD4+ T cells, Nef promotes the phosphorylation of WAVE2 by casein kinase 2 (CK2), thereby impairing WAVE2’s ability to activate the Arp2/3 branch of actin nucleation. This induces severe lamellar foot abnormalities in CD4+ T cells, leading to impaired immune function and dysfunction (Dunn et al., 2026). This provides a new mechanism for HIV-mediated CD4+ T cell dysfunction and exhaustion. Additionally, Nef promotes viral immune evasion. Nef recruits the host p21-activated kinase 2 (PAK2) to inactivate cofilin, consequently stabilizing actin. At this point, the migration of infected T lymphocytes is restricted (Stolp et al., 2009). Recent studies have further revealed that the stabilization of actin by Nef prevents protein phosphatase 1 regulatory subunit 12C (R12C) from localizing to the RIG-I-like receptor member melanoma differentiation-associated protein 5 (MDA5) to dephosphorylate it (Laliberté et al., 2025). MDA5 requires R12C-mediated dephosphorylation to recognize viral RNA and thereby initiate the interferon response (Acharya et al., 2022). All of the above pathological effects occurred in *in vitro* experiments. In the clinical field, in recent years, the research mainly focused on that of He et al (He et al., 2019). They analyzed the peripheral blood CD4+ T cells of a large number of HIV-infected individuals and confirmed that cofilin would be over-activated in HIV patients. This led to the disruption of CD4+ T cell migration, chemotaxis, and immune function. The use of actin signaling by HIV is shown in **Figure 7**.

**Figure 7:**
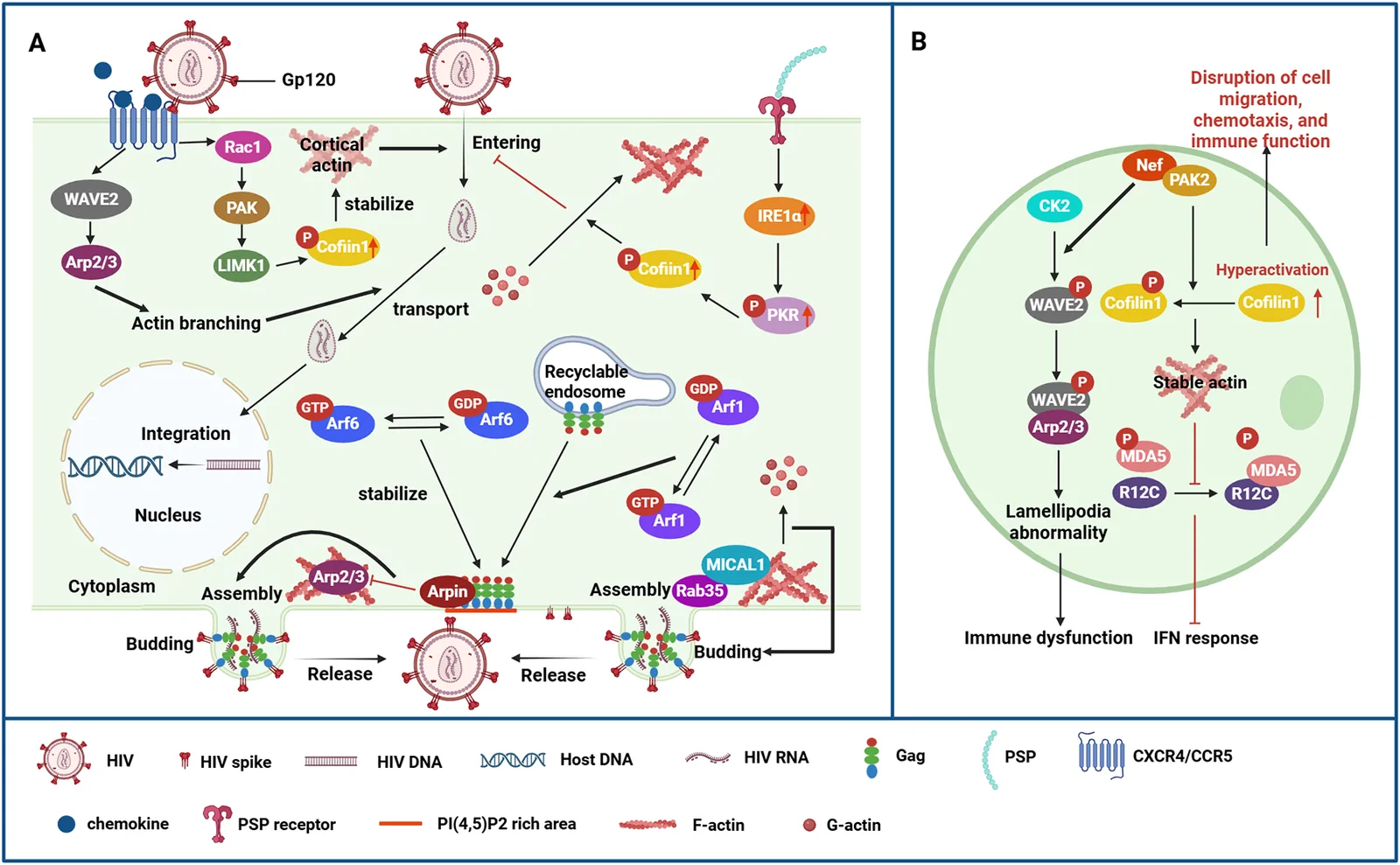
The interaction between HIV and the actin network. Black bold arrows indicate promotion. **(A)** Molecular mechanisms by which HIV hijacks actin during infection. In the early stage of viral infection, the virus binds to the CXCR4/CCR5 chemokine receptors, activating the Rac1-PAK-LIMK1-cofilin and WAVE2-Arp2/3-pathways. The dynamics of actin change, ultimately facilitating the entry of the virus and the integration of the genes. Concurrently, PSP promotes the activation of cofilin-1 by upregulating downstream signaling molecules, leading to actin polymerization and thereby inhibiting viral entry. During late viral infection, a dynamic shift in Arf1 activity facilitates the transport of the viral Gag protein to PI(4,5)P^2^-rich regions of the plasma membrane. Dynamic changes in Arf6 activity stabilize Gag on the plasma membrane. Gag subsequently recruits Arpin, and the Arp2/3 complex reduces actin branching, promoting virus assembly. During viral release, activation of MICAL1 by Rab35 leads to localized depolymerization of cortical actin, promoting viral budding and release. **(B)** Pathological consequences of HIV-mediated actin remodeling. After the viral Nef protein promotes the phosphorylation of WAVE2, the downstream Arp2/3 complex cannot be further activated, leading to abnormal lamellar feet and ultimately causing immune dysfunction. Additionally, Nef promotes the phosphorylation of cofilin1 to stabilize actin; at this point, R12C cannot phosphorylate MDA5, and the interferon response is suppressed. When cofilin is hyperactivated, multiple functions of T lymphocytes are also impaired. See the list of abbreviations at the end of the paper for the full meanings of all abbreviations. Created in BioRender. Jie, G. (2026) https://BioRender.com/4h980wq.

### 3.7 Others

In addition to the six representative RNA viruses mentioned above, there are several other viruses that cause disease in humans. The infection mechanisms by which these RNA viruses hijack actin are relatively simple and require further research to be better understood. Rotavirus (RV) is a major causative agent of severe acute gastroenteritis in children (Pawłuszkiewicz et al., 2025). Before RV invades a cell, its spike protein VP4 typically requires cleavage by proteases to produce the VP5* and VP8* fragments. A recent study has revealed that the K145-G150 loop in the VP8* fragment is a functionally critical region for VP4-mediated actin-dependent virion assembly. The presence of this loop promotes cortical actin reorganization, reduces stress fibers, and recruits Myosin IIa to the vicinity of the viral vesicle, ultimately facilitating efficient viral vesicle assembly (Vetter et al., 2022). Chikungunya virus (CHIKV) is an emerging mosquito-borne alphavirus that causes febrile illness and arthritis (Burt et al., 2017). During CHIKV infection, phosphorylated MAPK-activated protein kinase 2 (MK2) and MAPK-activated protein kinase 3 (MK3) further phosphorylate LIMK1, leading to the inactivation of cofilin. This promotes actin polymerization and the formation of pseudopods, facilitating the release of progeny viruses (Mamidi et al., 2021). CHIKV nonstructural protein 1 (nsP1) is the first nonstructural peptide cleaved from the viral polyprotein precursor; it can remodel the cell membrane through palmitoylation, thereby promoting filopodia formation (Zhang et al., 2019; Bakhache et al., 2020). Recent studies have further revealed that the Rac1-PAK1-Arp2 signaling axis is a key factor in nsP1-induced filopodia formation. This significantly promotes the efficient replication of the CHIKV genome (Aïqui-Reboul-Paviet et al., 2024). Nipah virus (NiV) is a zoonotic paramyxovirus with a case fatality rate ranging from 40% to 75% (Mazzola and Kelly-Cirino, 2019). NiV utilizes the interaction between its matrix protein and host F-actin to drive viral assembly and budding. Viral budding requires F-actin branching mediated by the Arp2/3 complex (Wang et al., 2025).

Similarly, infection by certain viruses can also trigger actin-mediated antiviral effects. Dabie bandavirus (DBV) is an emerging tick-borne pathogen that can cause multi-organ failure and even death in humans (Kim and Park, 2023). Liu et al. (2022) were the first to demonstrate that the small nonstructural protein (NSs) of DBV can induce actin reorganization by interacting with actin, thereby inhibiting viral adsorption and entry. This provides a unique target for antiviral proteins. Hantaan virus (HTNV) is a pathogen capable of causing hemorrhagic fever with renal syndrome. Guanylate-binding protein 1 (GBP1) is an interferon-stimulated gene that provides protection against infections by various pathogens (Cheng et al., 1983; Anderson et al., 1999). In addition, GBP1 has been shown to inhibit actin formation (Ostler et al., 2014). During HTNV infection, GBP1 can limit clathrin-mediated endocytosis (CME) to suppress viral infection. This may be because GBP1 specifically binds to actin, thereby hindering the recruitment of dynamin 2 to the CCP by actin (Gu et al., 2024). Mammalian orthoreovirus (MRV) is a zoonotic virus that typically causes mild disease in healthy individuals (Kobayashi, 2014). Annexin A2 (ANXA2) is a calcium-dependent phospholipid-binding protein capable of linking actin to the cell membrane (Chasserot-Golaz et al., 1996). During MRV infection, ANXA2 stabilizes the ER network by binding to long actin filaments. In the absence of ANXA2, the actin cytoskeleton tends to become disorganized and the endoplasmic reticulum undergoes remodeling; this accelerates VF formation during the early stages of viral infection and consequently increases viral yield (Tenorio et al., 2025). This indicates that ANXA2 is a key host factor that antagonizes MRV.

## 4 Potential antiviral compounds targeting actin signaling molecules

Given the high dependence of RNA viruses on actin cytoskeleton dynamics, targeting this host pathway has become a highly promising antiviral strategy. However, directly targeting actin itself often presents significant challenges, such as excessive cytotoxicity, a high degree of redundancy in physiological processes, and considerable heterogeneity among cell types (Bryce et al., 2019; Cho and Kim, 2026). Therefore, small molecules that regulate upstream signaling nodes are gradually emerging as an important branch of host-directed antiviral approaches. In recent years, with the in-depth analysis of the regulatory network governing actin dynamics, a series of small-molecule compounds targeting actin have been discovered and validated. By specifically interfering with actin signaling nodes, these compounds manifest substantial antiviral activity at the cellular level. In recent years, antiviral targets in the actin pathway can be generally classified into the following three categories (see **Table 2**).

**Table 2:** Anti-RNA viral compounds targeting actin. See the list of abbreviations at the end of the paper for the full forms of all abbreviations.

Compound	Target type	Target protein or pathway	Corresponding RNA virus	Year	Ref.
CK869	ABPs	Arp2/3	SARS-CoV-2	2022	(Endo et al., 2022)
Imatinib		C-ABL	DENV	2025	(Carreño-Flórez et al., 2025)
Vacuolin-1		CapZ	DENV, ZIKA	2024	(Zhu et al., 2024)
Melatonin	Rho GTPases	RhoA	DENV-4, ZIKV	2025	(Bravo-Silva et al., 2025)
phthFGL		RhoA-ROCK	DENV-2	2025	(Roa-Linares et al., 2025)
FX06		RhoA-ROCK1	SARS-CoV-2	2025	(Wang et al., 2025)
Atorvastatin, Simvastatin		Rac1	RSV	2021	(Malhi et al., 2021)
ZCL278		Cdc42	DENV, JUNV	2016	(Chou et al., 2016)
PIK-24	Upstream Proteins	PI3K-Cdc42-Cofilin	RSV	2022	(Chen et al., 2022)
ESI-09		EPAC-Rac1, EPAC-Cdc42	SARS-CoV-2	2023	(Boulton et al., 2023)
OTSSP167		MELK-Cofilin	Coronavirus	2026	(Yu et al., 2026)

### 4.1 Targeting ABPs

CK869 is an ARP2/3 complex inhibitor that inhibits branched actin nucleation by inserting into the hydrophobic core of the Arp3 subunit (Nolen et al., 2009). In SARS-CoV-2-infected cells, CK869 alone can reduce ACE2 expression to some extent. However, when used in combination with the 5-(N-ethyl-N-isopropyl)amiloride (EIPA) inhibitor, there was a significant downregulation of ACE2 protein expression on the cell surface. Furthermore, this combination also inhibits ACE2-independent entry pathways of the virus (Endo et al., 2022). This suggests that CK869 may disrupt the cytoskeleton, thereby simultaneously blocking multiple potential entry routes for the novel coronavirus. Imatinib is the world’s first commercially available oral multitarget tyrosine kinase inhibitor (Heinrich et al., 2000). During DENV infection, imatinib blocks actin remodeling by inhibiting c-ABL kinase activity, thereby preventing viral entry into cells via laminin-mediated endocytosis (Wu et al., 2015; Carreño-Flórez et al., 2025). Vacuolin-1 has been identified as a specific small molecule that targets CapZβ by inhibiting endosomal transport and trafficking through its binding to CapZβ[ (Ye et al., 2021). By stabilizing CapZ, Vacuolin-1 prevents the transition of early endosomes (EEs) to late endosomes (LEs) during infection by RNA viruses including DENV and ZIKA. This ultimately inhibits the viral infection process (Zhu et al., 2024).

### 4.2 Targeting Rho GTPases

#### 4.2.1 RhoA pathway

Melatonin is a neurohormone primarily secreted by the pineal gland that possesses immunomodulatory and neuroprotective properties (Maestroni et al., 1986; Ozacmak et al., 2009). During ZIKV and DENV-4 infections, melatonin treatment reduces RhoA activity, which, in turn, substantially reduces viral load of ZIKV and DENV-4 (Bravo-Silva et al., 2025). This reduction in RhoA activity may stabilize the actin cytoskeleton. The ferruginol analog 18-(phthalimid-2-yl)ferruginol (phthFGL) is a semi-synthetic analog of a bioactive diterpenoid ferruginol; molecular docking studies have confirmed that it binds with extremely high affinity near the active site of RhoA, thereby inhibiting RhoA activity. Consequently, treatment of DENV-2-infected cells with phthFGL destroys the actin filaments formed via RhoA-ROCK-mediated signaling. For DENV, this directly interferes with the physical platform required for the assembly of its replication complex, as well as the transport routes for viral components within the cell (Roa-Linares et al., 2025). SARS-CoV-2 infection leads to elevated levels of cytokines and chemokines, resulting in the elongation of F-actin stress fibers and increased angiogenesis. However, the fibrin-derived natural peptide FX06 significantly attenuates this effect. FX06 downregulates the expression of its downstream phosphorylated protein, ROCK1, by inactivating RhoA, eventually reducing angiogenesis (Wang et al., 2025).

#### 4.2.2 Rac1 pathway

Atorvastatin is the most widely prescribed statin worldwide; studies have shown that it also inhibits the downstream production of lipid isoprenoid intermediates (Blanco-Colio et al., 2002; Cicha et al., 2004). Simvastatin is another statin; when combined with atorvastatin, it markedly diminishes isopentenylation of Rac1 during RSV infection. This leads to reduced Rac1 activity, ultimately inhibiting viral infection (Malhi et al., 2021). NSC23766 is a specific Rac1 inhibitor. By binding to the specific binding groove for guanylate exchange factors on the surface of Rac1, NSC23766 blocks the interaction between Rac1 and GEFs (Gao et al., 2004). Although these Rac1-targeted drugs all possess antiviral activity, their effects on actin dynamics remain to be investigated.

#### 4.2.3 Cdc42 pathway

Compared to research on antiviral drugs targeting the RhoA and Rac1 pathways, progress on small-molecule compounds targeting Cdc42 against RNA viruses has been extremely limited in recent years. Currently, the classic tool molecule ZCL278 remains the sole core research vehicle for this pathway. ZCL278 is a highly selective small-molecule inhibitor of Cdc42 that specifically blocks Cdc42-mediated local actin remodeling. Early studies have confirmed that it exhibits significant inhibitory activity against the invasion of various RNA viruses, including DENV and Junin virus (JUNV), and can effectively block viral cell membrane anchoring and intracellular transport (Chou et al., 2016). However, whether the ultimate antiviral effect of ZCL278 involves actin remains to be further verified.

### 4.3 Targeting other upstream regulatory proteins

In addition to the ABP and Rho GTPase pathways, several antiviral compounds targeting more upstream nodes of the actin signaling pathway have been identified in recent years. As mentioned earlier, RSV relies on activation of the PI3K-mediated Cdc42-cofilin pathway to promote actin cytoskeleton reorganization and syncytial formation. The PI3K inhibitor PIK-24 can reverse this process by targeting the p85α regulatory subunit of PI3K, thereby exerting antiviral activity (Chen et al., 2022). ESI-09 is a selective inhibitor of exchange protein activated by cAMP (EPAC), which is often involved in actin remodeling during viral entry (Sehrawat et al., 2008). Recent studies have found that ESI-09 exhibits antiviral activity against SARS-CoV-2. The underlying molecular mechanism likely involves EPAC inhibition by ESI-09, which subsequently inhibits downstream Rac1 and Cdc42. In turn, the inhibition of Rac1 activates RhoA, resulting in increased formation of actin stress fibers (Boulton et al., 2023). OTSSP167 is a highly selective, small-molecule ATP-competitive inhibitor of maternal embryonic leucine zipper kinase (MELK). During various coronavirus infections, OTSSP167 blocks MELK, leading to dephosphorylation of cofilin-1. This leads to F-actin depolymerization, ultimately blocking coronavirus endocytosis and late-stage replication steps (Yu et al., 2026).

## 5 Conclusion and outlook

RNA viruses such as SARS-CoV-2 remain major pathogens threatening global public health. The actin cytoskeleton functions as a key host tool that facilitates viral infection. This article reviews advances in the molecular mechanisms by which actin facilitates infection by human RNA viruses, while also assessing the prospect of targeting actin regulatory factors to develop novel antiviral therapies. The current refinement of the actin regulatory networks hijacked by RNA viruses helps us identify additional antiviral targets. In-depth research on these targets will provide important insights concerning clinical pharmacology.

The core regulatory factors of actin are primarily ABPs and Rho GTPases. It was previously believed that different ABPs were responsible for their respective functional modules; however, recent discoveries have demonstrated the complexity of ABP functions. For example, while the tropomodulin family typically seals the minus end of actin filaments, one of its members, Lmod2, is capable of extending the minus end. Similar to ABPs, the factors mediating Rho GTPase activity also show complex regulatory mechanisms. These new findings further improve our understanding of the actin regulatory network. During RNA viral infection, viruses specifically hijack signaling pathways mediated by ABPs and Rho GTPases to regulate actin dynamics. Viral infection also induces other upstream signaling responses, including the PI3K-AKT pathway, which act as signaling bridges between the virus and Rho GTPases/ABPs. Furthermore, actin can inhibit viral infection. For example, TNF and IL-1 promote actin bundling by targeting the RhoA-ROCK pathway, ultimately inhibiting cell fusion. This further elaborates the relationship between actin and RNA viruses. Research on antiviral small-molecule drugs targeting the aforementioned signaling pathways has now achieved significant progress. Some of these compounds are repurposed drugs, while others are newly developed. However, all have demonstrated strong potential for antiviral activity.

Although the prospects for combating RNA viruses are promising, various important issues remain to be addressed. It is currently known that core signaling cascades, such as Rho GTPases, are hijacked by viruses to regulate the actin cytoskeleton. However, the upstream activation signals and downstream feedback loops of these molecules have not yet been fully elucidated. At the same time, regulatory factors newly discovered in recent years, such as NUAK2, have not yet been fully explored. Future research needs to be more comprehensive to map the actin signaling networks exploited by RNA viruses fully. Furthermore, targeting actin has always presented some intractable challenges. Actin is a highly conserved housekeeping protein, and directly targeting F-actin with classic tool drugs such as cytochalasin D indiscriminately disrupts fundamental cellular physiological processes. This leads to severe off-target cytotoxicity and an extremely narrow therapeutic window (Bryce et al., 2019). Second, the dynamic changes in the actin network exhibit a high degree of cell-type heterogeneity and specificity to different stages of the viral life cycle. The same drug may exhibit diametrically opposed effects in different cell types or at different stages of infection. This greatly increases the difficulty of drug design. Although in recent years, antiviral targets have gradually shifted from actin itself to the actin regulatory networks hijacked by viruses, these regulatory factors often simultaneously participate in processes such as the host’s immune response. Future efforts should focus more on virus-specifically hijacked actin regulatory nodes, achieving selective intervention by inhibiting specific interaction interfaces between viral proteins and host cytoskeletal proteins (such as the binding site between the RSV M protein and palladin). At the same time, multi-omics technologies and live-cell imaging can be utilized to map high-resolution actin network profiles at different stages of various viral infections, thereby developing drugs based on stage-specific activation. In terms of drug development, most drug candidates have only undergone *in vitro* validation, and research on novel derivatives and combination therapy regimens remains severely lacking. Future efforts should focus on rational molecular redesign to enhance pathway-specific targeting and reduce host cell toxicity. Optimizing drug delivery systems and developing next-generation candidate molecules will help advance the clinical translation of host-targeted, broad-spectrum anti-RNA virus strategies (Akalu et al., 2025; De Carli et al., 2025).

## Glossary

ABP: actin-Binding Protein
ACE2: angiotensin-converting enzyme 2
ADD1: adducin 1
AIP1: actin-interacting protein 1
AJC: apical junctional complex
AKT: protein kinase B
ANXA2: annexin A2
Arf1: ADP-ribosylation factor 1
Arf6: ADP-ribosylation factor 6
Arp2/3: actin-related protein 2/3
c-ABL: cellular abelson tyr kinase
CAPG: macrophage capping protein
CapZ: actin capping protein
CCTN: cortactin
CCS: clathrin-coated structure
CCP: clathrin-coated pit
Cdc42: cell division control protein 42 homolog
CHIKV: chikungunya virus
CME: clathrin-mediated endocytosis
CPs: capping proteins
CXCR3: C-X-C motif chemokine receptor 3
DBV: dabie bandavirus
DENV: dengue virus
DUSP5: dual specificity phosphatase 5
dsRNA: double-stranded RNA
EBOV: ebola virus
EE: early endosome
ENV: envelope protein
ERK: extracellular signal-regulated kinase
ERM: Ezrin/Radixin/Moesin
FBP17: formin-binding protein 17
FLNa: filamin A
FLNb: filamin B
FLNc: filamin C
F-Actin: filamentous actin
G-Actin: globular actin
GAP: GTPase-activating Protein
GBP1: guanylate-binding protein 1
GEFs: guanine nucleotide exchange factors
GP: glycoprotein
HA: hemagglutinin
HIV: human immunodeficiency virus
Hmpv: human metapneumovirus
HTNV: hantaan virus
IAV: influenza A virus
IL-1: interleukin-1
IL-1β: interleukin-1 beta
IL-1R1: interleukin-1 Receptor 1
IRE1α: inositol-requiring protein 1alpha
IP-10: interferon-gamma-inducible protein 10
IRAK1/4: interleukin-1 receptor-associated kinase ¼
JEV: Japanese encephalitis virus
JUNV: junin virus
LARG: leukemia-associated RhoGEF
LIMK: LIM domain kinase
Lmod2: leiomodin-2
M: matrix protein
MAPK: mitogen-activated protein kinase
MDA5: melanoma differentiation-associated protein 5
MICAL1: molecule interacting with CasL-1
MK2: MAPK-activated protein kinase 2
MK3: MAPK-activated protein kinase 3
MLCK: myosin light chain kinase
MLC: myosin light chain
MLC2: myosin regulatory light chain 2
MRV: mammalian orthoreovirus
MyD88: myeloid differentiation primary response protein 88
NiV: nipah virus
nsP1: nonstructural protein 1
NSs: small nonstructural protein
NUAK2: NUAK family kinase 2
PAK: p21 activated kinase
PAK2: p21 activated kinase 2
PIC: preintegration complex
PI3K: phosphoinositide 3-kinase
PI3K p85α: PI3K p85α regulatory subunit
PI(3)P: phosphatidylinositol 3-phosphate
PI(4,5)P₂: phosphatidylinositol 4,5-bisphosphate
PI(3,5)P₂: phosphatidylinositol 3,5-bisphosphate
PKC: protein kinase C
PKN: protein kinase N
PKR: double-stranded RNA-dependent protein kinase
PRG: PDZ domain-containing Rho GEF
PSP: polysaccharopeptide
R12C: regulatory subunit 12C
Rab5: ras-related protein Rab-5A
Rab7: ras-related protein Rab-7a
Rab11a Res: rab11a-positive recycling endosomes
Rab35: ras-related protein rab-35
RABV: rabies virus
Rac1: ras-related C3 botulinum toxin substrate 1
Rap1: ras-related protein 1
RBD: receptor binding domain
RhoA: ras homologous A
RIPK1: receptor-interacting serine/threonine-protein kinase 1
ROCK: Rho-associated protein kinase
RSV: respiratory Syncytial virus
RV: rotavirus
SARS-CoV-2: severe acute respiratory syndrome coronavirus 2
SDC4: syndecan-4
SM: sphingomyelin
SSH3: phosphatases slingshot 3
TRADD: TNFR1-associated death domain protein
TRAF2: TNF receptor-associated factor 2
TRAF6: TNF receptor-associated factor 6
TNFR1: tumor necrosis factor receptor 1
TNF: tumor necrosis factor
TPECs: thioredoxin fold-containing pointed-end capping proteins
UPR: unfolded protein response
VF: viral factory
VSV: vesicular stomatitis virus
vRNPs: viral ribonucleoprotein complexes
WASP: wiskott–Aldrich syndrome protein
WAVE: WASP family Verprolin-homologous protein
-ssRNA: negative-sense single-stranded RNA
+ssRNA: positive-sense single-stranded RNA
